# Supplementary material for: Evaluation of the Effect of Adipokinetic Hormone/Corazonin-Related Peptide (ACP) on Ovarian Development in the Mud Crab, Scylla paramamosain
Source: Animals (Basel). 2024 Dec 23;14(24):3706. doi: 10.3390/ani14243706 (PMC11672810; doi:10.3390/ani14243706)
Supplement: Supplementary file 1 [file animals-14-03706-s001.zip › animals-3358910-supplementary.pdf]

Supplemental Table S1

Table S1. Summary of primers used in this study.

Primer	Primer sequence (5′-3′)	Application
ACP-F	ATGGCGAGCTGGATGTTGG	cDNA cloning
ACP-R	GTGTGACTGGCTTTAGGGAAGC	cDNA cloning
ACPR-F	ATGTCAGTGGTGCCAAGGACAGGA	cDNA cloning
ACPR-R	TTAGGGGGAGGAGGGAAGGG	cDNA cloning
ACP-QF	CAGCGTCACTCCTCAGATCA	qRT-PCR
ACP-QR	CTTACCAAGAGGTGACCAGCA	qRT-PCR
ACPR-QF	CGTCGGGAACCTACTGGTGT	qRT-PCR
ACPR-QR	AACGCCGACAGGAACTTGTA	qRT-PCR
Vg-QF	CGCAACCGCCACTGAAGAT	qRT-PCR
Vg-QR	CCACCATGCTGCTCACGACT	qRT-PCR
β-actin-QF	GAGCGAGAAATCGTTCGTGAC	qRT-PCR
β-actin-QR	GGAAGGAAGGCTGGAAGAGAG	qRT-PCR
VgR-QF	TTCTATACCAGGCCACTACC	qRT-PCR
VgR-QR	TTTTCACTCCAAGCACACTC	qRT-PCR
